# Comparison of parasite load by qPCR and histopathological changes of inner and outer edge of ulcerated cutaneous lesions of cutaneous leishmaniasis

**DOI:** 10.1371/journal.pone.0243978

**Published:** 2021-01-21

**Authors:** Caio Thomaz, Cintia Xavier de Mello, Otávio de Melo Espíndola, Armando de Oliveira Shubach, Leonardo Pereira Quintella, Raquel Vasconcelos Carvalhaes de Oliveira, Adriane Corrêa Gomes Duarte, Maria Inês Fernandes Pimentel, Marcelo Rosandiski Lyra, Mauro Celio de Almeida Marzochi

**Affiliations:** 1 Laboratório Interdisciplinar de Pesquisas Médicas, Instituto Oswaldo Cruz (IOC), Fundação Oswaldo Cruz, Rio de Janeiro, RJ, Brasil; 2 Laboratório de Pesquisa Clínica e Vigilância em Leishmanioses, Instituto Nacional de Infectologia Evandro Chagas (INI), Fundação Oswaldo Cruz, Rio de Janeiro, RJ, Brasil; 3 Laboratório de Pesquisa Clínica em Neuroinfecções, Instituto Nacional de Infectologia Evandro Chagas (INI), Fundação Oswaldo Cruz, Rio de Janeiro, RJ, Brasil; 4 Serviço de Anatomia Patológica, Instituto Nacional de Infectologia Evandro Chagas (INI), Fundação Oswaldo Cruz, Rio de Janeiro, RJ, Brasil; 5 Laboratório de Epidemiologia Clínica, Instituto Nacional de Infectologia Evandro Chagas (INI), Fundação Oswaldo Cruz, Rio de Janeiro, RJ, Brasil; Netherlands Cancer Institute, NETHERLANDS

## Abstract

**Background:**

Cutaneous leishmaniasis (CL) is an infectious vector-borne disease caused by protozoa of the *Leishmania* genus that affects humans and animals. The distribution of parasites in the lesion is not uniform, and there are divergences in the literature about the choice of the better sampling site for diagnosis–inner or outer edge of the ulcerated skin lesion. In this context, determining the region of the lesion with the highest parasite density and, consequently, the appropriate site for collecting samples can define the success of the laboratory diagnosis. Hence, this study aims to comparatively evaluate the parasite load by qPCR, quantification of amastigotes forms in the direct exam, and the histopathological profile on the inner and outer edges of ulcerated CL lesions.

**Methods:**

Samples from ulcerated skin lesions from 39 patients with confirmed CL were examined. We performed scraping of the ulcer inner edge (base) and outer edge (raised border) and lesion biopsy for imprint and histopathological examination. Slides smears were stained by Giemsa and observed in optical microscopy, the material contained on the smears was used to determine parasite load by quantitative real-time PCR (qPCR) with primers directed to the *Leishmania (Viannia)* minicircle kinetoplast DNA. The histopathological exam was performed to evaluate cell profile, tissue alterations and semi-quantitative assessment of amastigote forms in inner and outer edges.

**Principal findings:**

Parasite loads were higher on the inner edge compared to the outer edge of the lesions, either by qPCR technique (*P*<0.001) and histopathological examination (*P*< 0.003). There was no significant difference in the parasite load between the imprint and scraping on the outer edge (*P* = 1.0000).

**Conclusion/Significance:**

The results suggest that clinical specimens from the inner edge of the ulcerated CL lesions are the most suitable for both molecular diagnosis and direct parasitological examination.

## Introduction

Cutaneous leishmaniasis (CL) is an infectious disease, caused by protozoa of the *Leishmania* genus, transmitted by sandflies bite [1]. CL is widespread in tropical and subtropical areas and about 95% of new cases mainly occur in 6 countries, including Brazil [2]. In Brazil, CL is associated with seven different species of *Leishmania*, being *Leishmania* (*V*.) *braziliensis* the most prevalent species [3].

The typical ulcer of CL is, in most cases, unique, rounded, painless, measuring up to a few centimeters, with an infiltrated and hardened base, well defined, elevated and erythematous edges and a granular and reddish bottom [3].

The histopathological examination is a diagnostic method that, despite having low sensitivity for viewing amastigote forms, is important in the differential diagnosis and for allowing the study of the inflammatory infiltrate associated with infection by parasites of the *Leishmania* genus. Histopathological changes in CL can be seen in histological sections stained with hematoxylin and eosin. Such changes are characterized by a chronic inflammatory reaction, usually granulomatous, with the presence of lymphocytes, plasma B cells and macrophages. There may also be focal necrotic areas [4, 5].

The direct parasitological diagnosis, through microscope visualization of amastigote forms, presents low sensitivity, especially in chronic CL due to infections by *L*. (*V*.) *braziliensis*, characterized by low parasite load in the lesions [6, 7]. The traditional methods of obtaining samples for the direct examination are the imprint and scraping. A biopsy of the lesion is carried out to prepare the imprint, and the collected tissue fragment is lightly pressed on a microscopic slide. This method has the disadvantage of including anesthesia steps, surgical excision of the tissue fragment and suture which are procedures exclusive to the medical professional.

In contrast, scraping is a less invasive procedure that can be performed by technical professionals equipped with scalpel or lancet blades, without needing anesthesia [3, 8]. According to World Health Organization recommendations, the samples must be collected from the edge of the ulcerated skin lesion, as it is assumed that this site contains a more significant amount of parasites. The Ministry of Health in Brazil is more specific and recommends the collection of specimens from the internal edge of the lesion [2, 3]. Some studies using qPCR have shown a higher parasite load in the inner edge or even in the center of the lesion [9, 10].

Clinical specimens have to be collected directly from cutaneous lesions so establishing the most appropriate sampling site is essential to an accurate diagnosis. To this end, we proposed to evaluate comparatively the parasite load by qPCR, direct exam for amastigotes forms and histopathological analysis in inner edge (IE) and outer edge (OE) of ulcerated skin lesions of cutaneous leishmaniasis.

To our knowledge, this is the first study that compares by histopathological examination, cell types, histopathological changes, and the number of amastigote forms of *L*.*(V*.*)*.*braziliensis* in the inner and outer edge of CL ulcerated lesion, which associated to parasite loads quantified by qPCR is important to better understand parasite distribution within the lesion.

## Methods

### Ethical aspects

The Research Ethics Committee of the National Institute of Infectious Diseases Evandro Chagas (INI/Fiocruz) approved this study (project code: 96556718.9.0000.5262). All patients attended at Clinical Research and Surveillance Laboratory for Leishmaniasis (INI / Fiocruz) were given information and explanation of the purposes of the research and the consent form. All patients included in this study agreed to participate by signing an informed consent form.

### Study design and research participants

This is a cross-sectional analysis to assess parasite load in samples obtained by scraping the IE (base) and OE (raised border) and by imprinting in ulcerated CL lesion. Population consisted of a cohort of 39 patients, over 18 years old, with ulcerated lesion, with a confirmed diagnosis of CL and treated at the Clinical Research and Surveillance Laboratory for Leishmaniasis (INI / Fiocruz), Rio de Janeiro, Brazil, during 2009 and 2010. In all cases, according to a previous study, the diagnosis was established by culture isolation and such parasites were characterized as *L*. (*V*.) *braziliensis* [11].

In all patients, samples were collected both by scraping and by biopsy on the edge of the lesions.

The information regarding the variables: date of birth, sex, probable location of the infection, time of lesion evolution, and location of the lesion were retrieved from the patients' medical records.

### Diagnostic and laboratory procedures

#### Imprint and scraping preparation

**Fig 1:**
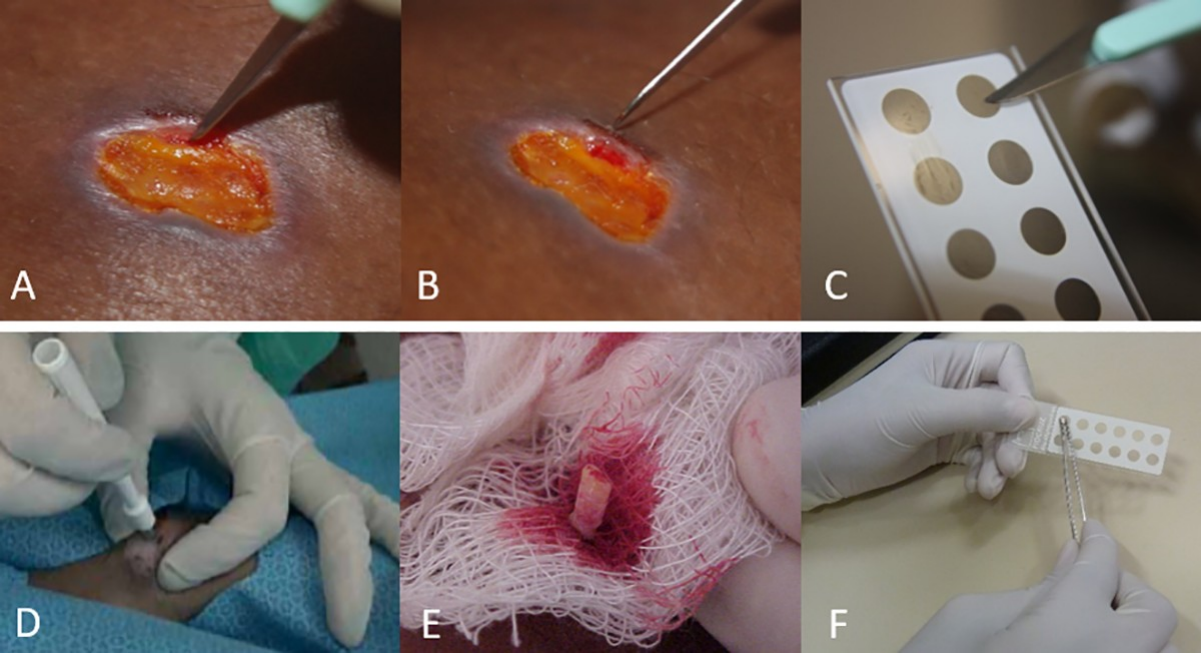
Sampling of clinical specimens from patients with cutaneous leishmaniasis. **A**: Scraping of the inner edge of the lesion with the aid of scalpel; **B**: Scraping of the outer edge of the lesion with the aid of scalpel **C**: Distension of the material in a microscopy slide with delimited areas; **D**: Biopsy with 5mm punch; **E**: Removal of excess blood from the fragment tissue; **F**: Imprint prepared with fragment tissue on the slide. Source: Clinical Research and Surveillance Laboratory for Leishmaniasis.

For each patient was performed a scraping at the IE and OE with a sterile scalpel and the samples collected were put in two microscopic slides (Fig 1). For histopathological examination and imprint, a biopsy was performed at the previously scraping site, with the aid of a punch (5mm), to collect a tissue fragment containing 1/3 of ulcer inner edge and 2/3 of the outer edge of the lesion. For lesion smears it was used microscope slides containing 12 delimited areas that were completely filled with material collected from the lesion [11]. A total of 156 slides were evaluated, 39 for each of the four tests (IE scraping, OE scraping, imprint, and histopathological examination).

#### Microscopic examination of scraping and imprint slides

The imprint and scraping slides were fixed with methanol, stained by Giemsa, and observed under an optical microscope in an immersion objective lens (x1000).

#### Semi-quantitative evaluation of scraping and imprint

For scraping and imprint preparations, a 12 delimited area microscopic slide was used. The presence of amastigotes in each of the delimited areas of the slide and the sum of positive areas were recorded during the microscopy reading. The results were compared among the slides of IE scraping, OE scraping, and imprint.

#### DNA extraction of outer and inner edge scraping and imprint slides

The slides were softly cleaned with tissue paper, and the entire length was scraped with the aid of a scalpel and sterile Petri dishes. The material was kept in a 1.5 mL polypropylene tube with proper identification. The extraction was performed using the ChargeSwitch™ Forensic DNA Purification Kit (Thermo Fisher Scientific) with the following protocol modifications: incubation with the reagents Lysis buffer and proteinase K at 55°C "overnight" and final elution volume of 50μL. The isolated and purified DNA was quantified by fluorimetry using the Qubit Fluorimeter 3.0 (Thermo Fisher Scientific).

### Quantitative assessment of parasite load

#### Standard curve

Reference strain of *Leishmania braziliensis* (MHOM / BR / 1975 / M2903) was used. The parasites were inoculated in Schneider’s Drosophila medium (Sigma Chemical Co., USA) supplemented with 20% fetal bovine serum (Life Technologies, Brazil), L-glutamine (1 mM; Life Technologies, Brazil)), and antibiotics (200 U/mL penicillin and 200 μg / mL streptomycin) and then placed in an incubator at 27°C. At the fourth day parasites were counted in a Neubauer chamber and DNA was extracted from 1.0x10^7^ parasites using the Qiagen DNeasy Blood & Tissue Kit® (Qiagen), according to the manufacturer's recommendations, and eluted in 100μL. The DNA concentration obtained from the extraction was 9.1 ng/μL determined using the Qubit Fluorimeter^®^. The *Leishmania* standard curve was built from 10-fold serial dilutions ranging from 1.0 x10^4^ to 1.0 x10^-2^ parasite DNA equivalents/reaction [12].

The curve was validated by three independent reactions in triplet of each concentration. An arithmetic average and the standard deviation of the quantification cycles (Cqs) were performed in order to establish the Cqs of each dilution point and determine the linearity coefficient (R^2^) and efficiency of the standard curve.

#### Endogenous control of qPCR

All the samples were tested to exclude false-negative results due to low DNA extraction efficiency or the presence of PCR inhibitors. They were tested for the qualitative amplification of the β-actin gene using the primers described by Rodrigues et al., 2011 [13]. As a control of this reaction, we extracted DNA from 24 mg of fresh tissue, from a patient without cutaneous leishmaniasis, with the Qiagen DNeasy Blood & Tissue Kit® (Qiagen), according to the manufacturer's recommendations, with the final elution carried out in a volume of 100μL and then stored at -20°C. DNA amplification was performed using 5μL of PowerUp™ SYBR™ Green Master Mix (Thermo Fisher Scientific), 500nM of primer targeting the β-actin gene and 2.5μL of DNA in a total volume of 10μL.

#### Absolute quantification of parasite load

The primers used targeted the conserved region of the kDNA minicircle of *Leishmania* (*Viannia*) species [14]. The *Leishmania* standard curve was included in each run at concentrations ranging from 1.0x10^4^ to 1.0x10^-2^ parasite DNA equivalents/reaction in duplicate. Samples were diluted 1:5 and evaluated in triplets. Duplicate negative controls were used, “No Template Control” (NTC–just PCR reagents without DNA). DNA amplification was performed using 5μL of PowerUp™ SYBR™ Green Master Mix (Thermo Fisher Scientific), 300nM of primer targeting the kDNA target and 2.5μL of DNA in a total volume of 10μL. The thermocycler “StepOne® Real-Time PCR System” (Applied Biosystems) was used, and the amplification was performed according to the following cycle: 50°C for 2 minutes, 95°C for 2 minutes, 40 cycles of 95°C for 15 seconds and 60°C for 1 minute. At the end of each run, a melting curve analysis was performed in an initial denaturation stage at 95°C for 15s, followed by a decrease in temperature to 60°C for 1min and subsequent heating at 95°C for 15s to monitor primer-dimers or non-specific product formation. The calculation of the melting temperature of each amplicon was performed directly by the built-in software of the equipment.

The *Leishmania* parasite load was calculated as follows: [parasite DNA equivalents per reaction /amount of tissue DNA per reaction] x 10^3^, expressed as the number of *Leishmania* parasites per μg of tissue DNA [10].

Only those samples whose quantification cycle (Cq) values fell within the quantifiable range of the standard curve were considered. The highest dilution of template of the standard curve was defined as the limit of quantification.

#### Histopathological analysis

The tissue fragments were fixed in 10% neutral buffered formalin and processed according to the diagnostic routine of the Pathological Anatomy Service/INI/Fiocruz, briefly, paraffin embedding, 5 μm thick cuts and stained with hematoxylin-eosin. For histopathological analysis, samples selected were those in which the fragment could be oriented, and the outer and inner edges could be identified. For this study inner edge (ulcer base) was defined as a well-characterized ulcer (the replacement of the epithelial lining by exudate, and the presence of granulation tissue) as well and/or showing indicative changes of ulcerated lesion over the entire length of the sample in which such changes (pseudoepitheliomatous hyperplasia, necrosis or granulation tissue) were present, even if the epithelial lining was kept.

An optical microscope evaluation of the general histopathological characteristics and their changes were made for each sample [15]. A semi-quantitative analysis of the main cell types on the inner and outer edges was also performed. Additionally, on the examination with immersion oil (1000x magnification), a semi-quantitative evaluation adapted from a previous study [16] and a quantitative evaluation were performed in the microscope fields with the highest parasite density, considering the number of amastigote forms per microscope field and the parasitophorous vacuole in macrophages, on the inner and outer edges of the lesion.

### Analysis of results

The exploratory data analysis was performed by: (a) the enumeration of the frequencies and proportions to assess positivity in the delimited area; and (b) median areas and the interquartile range for the parasite load and total DNA. The normality of the variable parasite load was rejected by the Shapiro-Wilk test, which indicated the use of non-parametric tests. The Friedman test and Wilcoxon test with Bonferroni adjustment were performed to evaluate whether there were significant differences among the parasite loads quantified from the three sampling techniques (IE scraping, the OE scraping, and the imprint). It was also tested the differences among the distribution of the parasite loads according to evolution time (≤3 month and >3 months), the number of delimited positive areas by Kruskal Wallis and Mann-Whitney with Bonferroni adjustment tests. P-values <0.05 indicated statistically significant tests. Statistical analyses were performed using the GraphPad software 8.2.0 (GraphPad Software, Inc.).

## Results

### Patients

**Fig 2:**
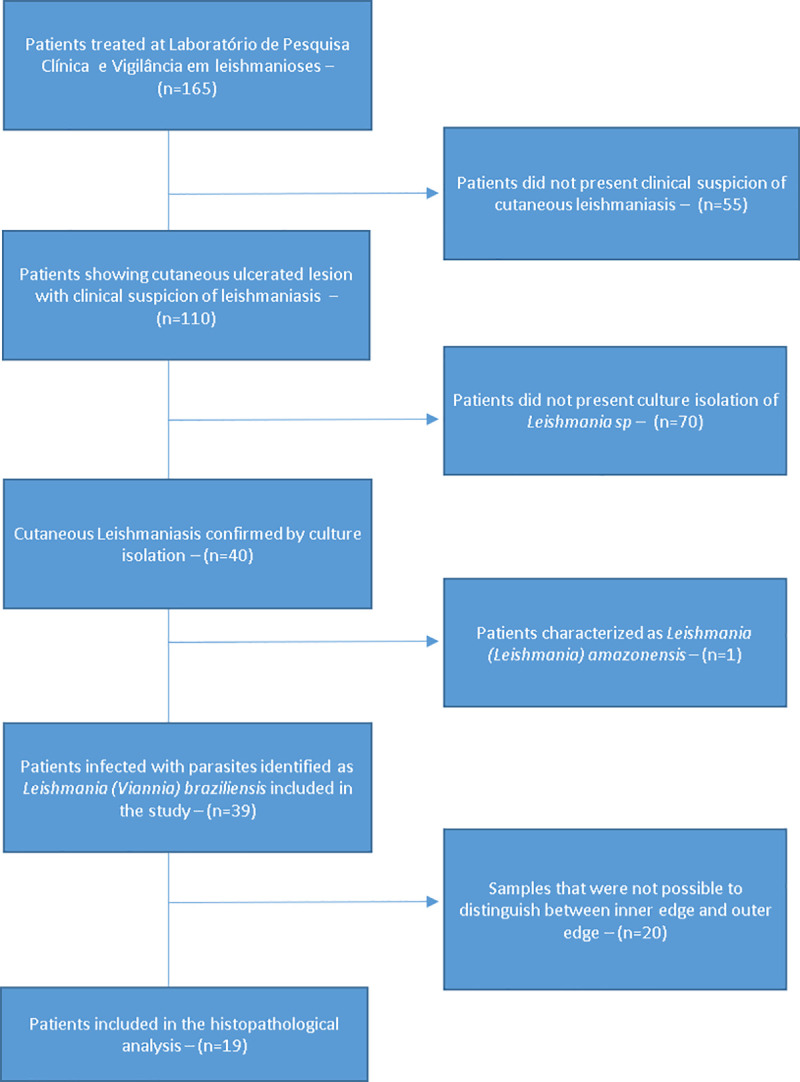
Flowchart of inclusion / exclusion of patients evaluated in the study, Rio de Janeiro, Brazil, 2009–2010. *16 cases had well characterized ulcers, 9 cases pseudoepitheliomatous squamous hyperplasia, 17 cases granulation tissue and 8 cases necrosis.

This study included 39 patients with a confirmed diagnosis of CL due to *Leishmania (Viannia) braziliensis* (Fig 2) which had sample collected from April/2009 to May/2010. Thirty-eight patients were from Rio de Janeiro state and 1 from Bahia state–Brazil. Sixty-nine percent were male. The median age was 44 years, the median of disease duration was 2 months and the median of number of lesions was 1. Regarding the lesion localization, 56,5% of patients presented lesions on arms, 12,8% in face/neck, 23% in legs and 7,7% in body.

Two patients (001 and 012 –S1 Table) presented relapsed lesions at the moment of sample collection. These detailed results for each patient are described in the S1 Table.

#### Direct exam semi quantitative evaluation

For each of the 117 samples (39 IE scraping slides, 39 OE scraping slides, and 39 imprint slides), the sum of positive delimited areas was counted in each type of direct exam.

**Fig 3:**
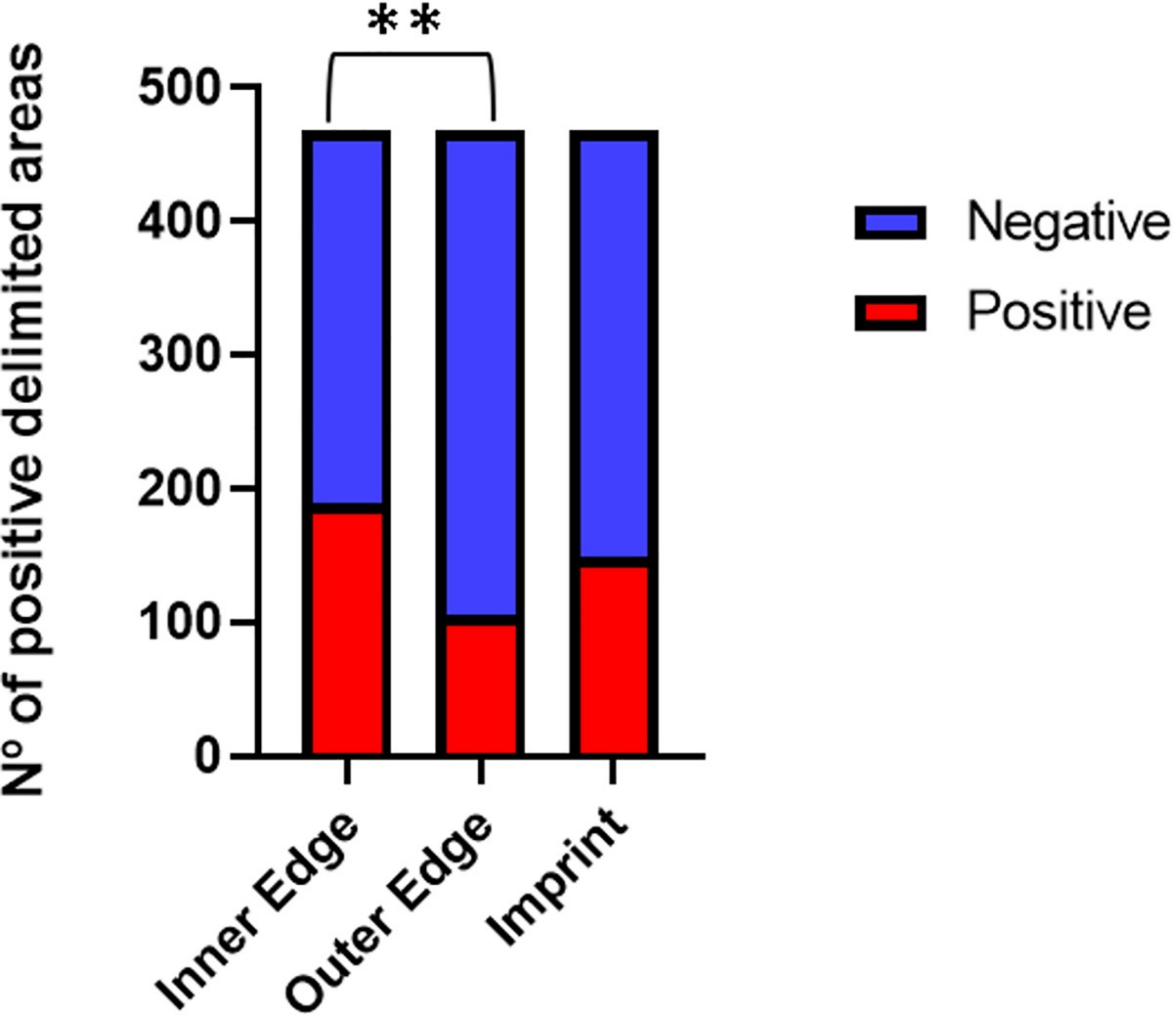
Number of positive delimited areas by direct examination in samples from scraping of inner and outer edge and imprint (x1000) of ulcerated skin lesions of cutaneous leishmaniasis in 39 patients, Rio de Janeiro, Brazil, 2009–2010. Friedman Test: *P =* 0,0092; Wilcoxon Test with Bonferroni correction: **P = 0,004.

From the evaluated slides, 23 positive results were obtained in the internal edge scraping, with a total of 189 positive delimited areas. In the external edge, 17 positive results were obtained, with 105 positive delimited areas. In the imprint, there were 27 slides positive, with 149 positive delimited areas. A higher number of positive delimited areas was found in the inner edge scraping of the skin ulcer compared with the outer edge scraping, which was statistically significant (Fig 3).

### Parasite load

During kDNA standard curve validation, we obtained an average efficiency of 97.47% [SD: 2.23], coefficient correlation (R^2^) of 0.995 [SD: 0.003] and slope of -3.385 [SD: 0.057]. A qualitative evaluation of β-actin gene, an internal control gene, was performed to exclude false-negative results due to low extraction efficiency or presence of inhibitors. The threshold value was set at 0.05. The melting temperature was 80.37°C [SD: 0.14] for kDNA and 83.94°C [SD: 0.13] for β-actin.

The fluorimetry quantification of the total DNA obtained after extraction ranged from 6.38 to 0.276 ng/μL for the inner edge (median: 1.542); 1.914 to 0.172 ng/μL for the outer edge (median: 0.612); and 4.34 to 0.004 ng/μL for imprint (median: 0.42).

It was possible to perform the absolute quantification of the parasite load of all 39 samples in scraping of the inner edge, the outer edge, and the imprint. All samples were positive in qPCR for β-actin. A higher parasite load was found in the inner edge scraping of the skin ulcer compared with the outer edge scraping and biopsy imprint, which was statistically significant.

**Fig 4:**
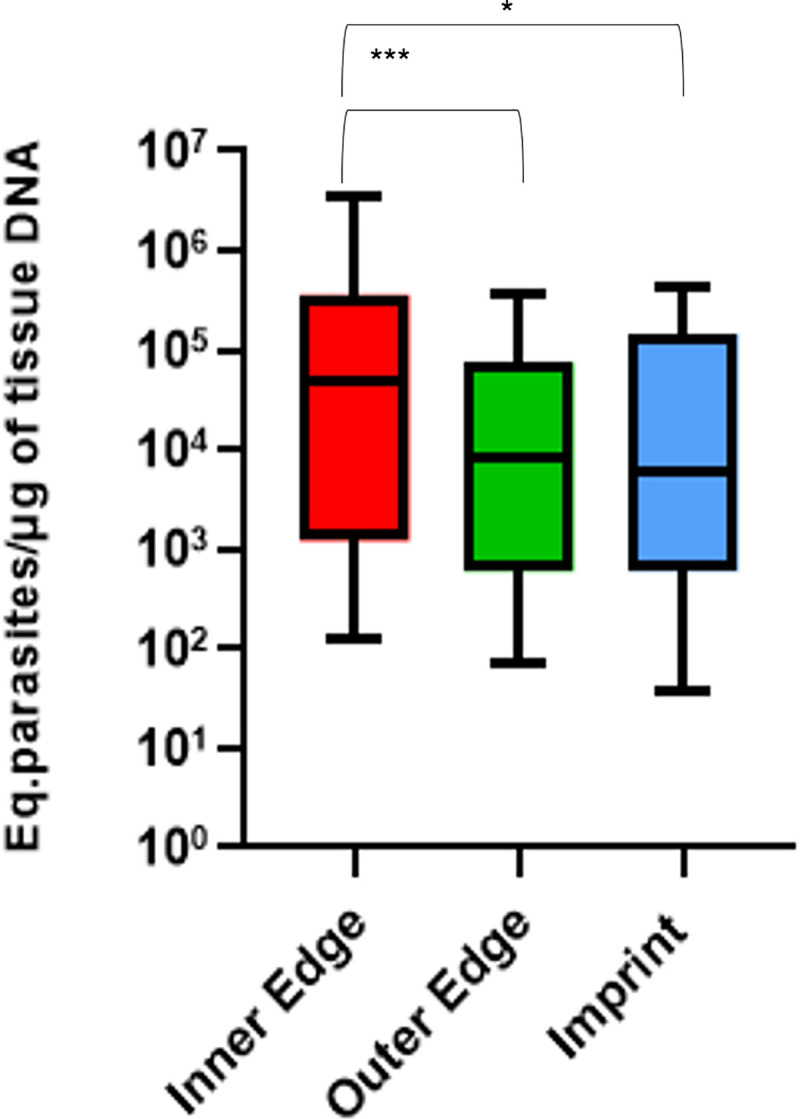
Absolute quantification of the parasite load in samples from imprint and scraping of the inner and outer edge of ulcerated skin lesions of 39 patients confirmed with CL, Rio de Janeiro, Brazil, 2009–2010. Friedman test P <0.001; Wilcoxon test with Bonferroni correction: * *P =* 0.029; *** *P<*0.001.

There was no significant difference between the parasite loads of outer edge scraping and biopsy imprint. There was no significant association between the parasite load levels quantified by qPCR and lesion evolution time. These results are shown in Table 1 and Fig 4.

**Table 1:** Parasite load levels of inner and outer edge scrapings and imprint of 39 patients with cutaneous ulcerated lesion of leishmaniasis. Rio de Janeiro–Brazil.

Lesion site	Parasite Load ^‡^								
	Lesion evolution ≤3 months (n = 26)			Lesion evolution >3 months (n = 13)			All patients (n = 39)		
	Median	Interquartile Range (25–75%)	Range of parasite load levels	Median	Interquartile Range (25–75%)	Range of parasite load levels	Median	Interquartile Range (25–75%)	Range of parasite load levels
**Inner Edge^¶^^,^^¥^**	7,59E+04	2,01E+03–3,47E+05	2,00E+00–3,42E+06	4,61E+03	1,20E+03–1,43E+05	1,30E+02–5,77E+06	4,91E+04	1,20E+03–3,60E+05	1,74E+00–5,77E+06
**Outer Edge^¶^**	1,12E+04	8,62E+02–7,31E+04	6,98E+01–3,65E+05	4,47E+03	9,47E+02–3,87E+04	3,60E+01–4,41E+06	8,61E+03	5,93E+02–7,54E+04	3,56E+01–4,41E+06
**Imprint^¥^**	1,57E+04	1,48E+03–1,37E+05	2,03E+01–7,89E+05	3,08E+03	8,09E+01–1,21E+04	3,85E+01–3,87E+05	5,93E+03	6,00E+02–1,46E+05	2,03E+01–7,89E+05

^‡^ Number of *Leishmania* parasites per μg of tissue DNA.

^¶^ *P*<0.001, for the comparison of parasite loads between inner and outer edges scraping (Friedman test and Wilcoxon test with Bonferroni correction).

^¥^ *P =* 0.029, for the comparison of parasite loads between inner edge and imprint (Friedman test and Wilcoxon test with Bonferroni correction).

### Histopathological analysis

The histopathological analysis was performed in 19 samples, for which it was possible to identify inner and outer edges of the ulcerated skin lesion. For all the analyzed characteristics, the same patient may show more than one histopathological aspect. In a sample of the outer edge, an association of well-formed and malformed granulomas was observed. One sample did not show granuloma. Four samples showed caseous necrosis concomitant with fibrinoid necrosis. These results are described in Table 2.

**Table 2:** Histopathological variables analyzed in fragments of ulcerated skin lesions from 19 patients with cutaneous leishmaniasis.

Histopathological findings			IE (n)	OE (n)
**Squamous Hyperplasia**	0	Absent	**5**	**3**
	+	Regular or Discreet	**3**	**14**
	++	Irregular	**2**	**2**
	+++	Pseudoepitheliomatous	**9**	**0**
**Exudative Cellular Reaction**	Unspecified inflammation		**1**	**1**
**Exudative Necrotic Reaction**	Unspecified inflammation with necrosis		**1**	**0**
**Exudative Granulomatous Reaction**	Granulomatous inflammation		**8**	**3**
**Exudative Necrotic and Granulomatous Reaction**	Granulomatous inflammation with necrosis		**18**	**18**
**Exudative Tuberculoid Reaction**	Tuberculoid granulomas (large, coalescent, with necrosis and Langerhans cells)		**4**	**2**
**Exudative Sarcoid Reaction**	Sarcoid granulomas (bare, rounded, cohesive)		**1**	**1**
**Well-formed granuloma**	Granuloma well delimited, rounded or oval, cohesive cells		**3**	**1**
**Malformed granuloma**	Poorly delimited granuloma, irregular shape, loose		**18**	**18**
**Types of Necrosis**	Caseous		**8**	**2**
	Liquefactive		**2**	**0**
	Coagulative		**0**	**1**
	Fibrinoid		**6**	**3**
	Other		**8**	**2**
**Plasmocytes**	0	Absent	**0**	**0**
	+	Small quantity in few microscopic fields	**0**	**2**
	++	Moderate quantity	**5**	**6**
	+++	Large amount in many fields	**10**	**7**
**Limphocytes**	0	Absent	**0**	**0**
	+	Small quantity in few microscopic fields	**3**	**4**
	++	Moderate quantity	**6**	**7**
	+++	Large amount in many fields	**6**	**4**
**Neutrophils**	0	Absent	**4**	**8**
	+	Isolated	**9**	**6**
	++	Small Abscesses	**2**	**1**
	+++	Great Abscesses	**0**	**0**
**Parasite index**	0	Absent	**5**	**8**
	+	Up to 05 amastigotes per standard cut (4 to 5 mm)	**3**	**4**
	++	From 06 amastigotes per standard cut to 05 per immersion field (x1000)	**1**	**1**
	+++	From 06 to 50 amastigotes per immersion field	**6**	**2**
	++++	More than 50 amastigotes per immersion field	**4**	**0**

**Fig 5:**
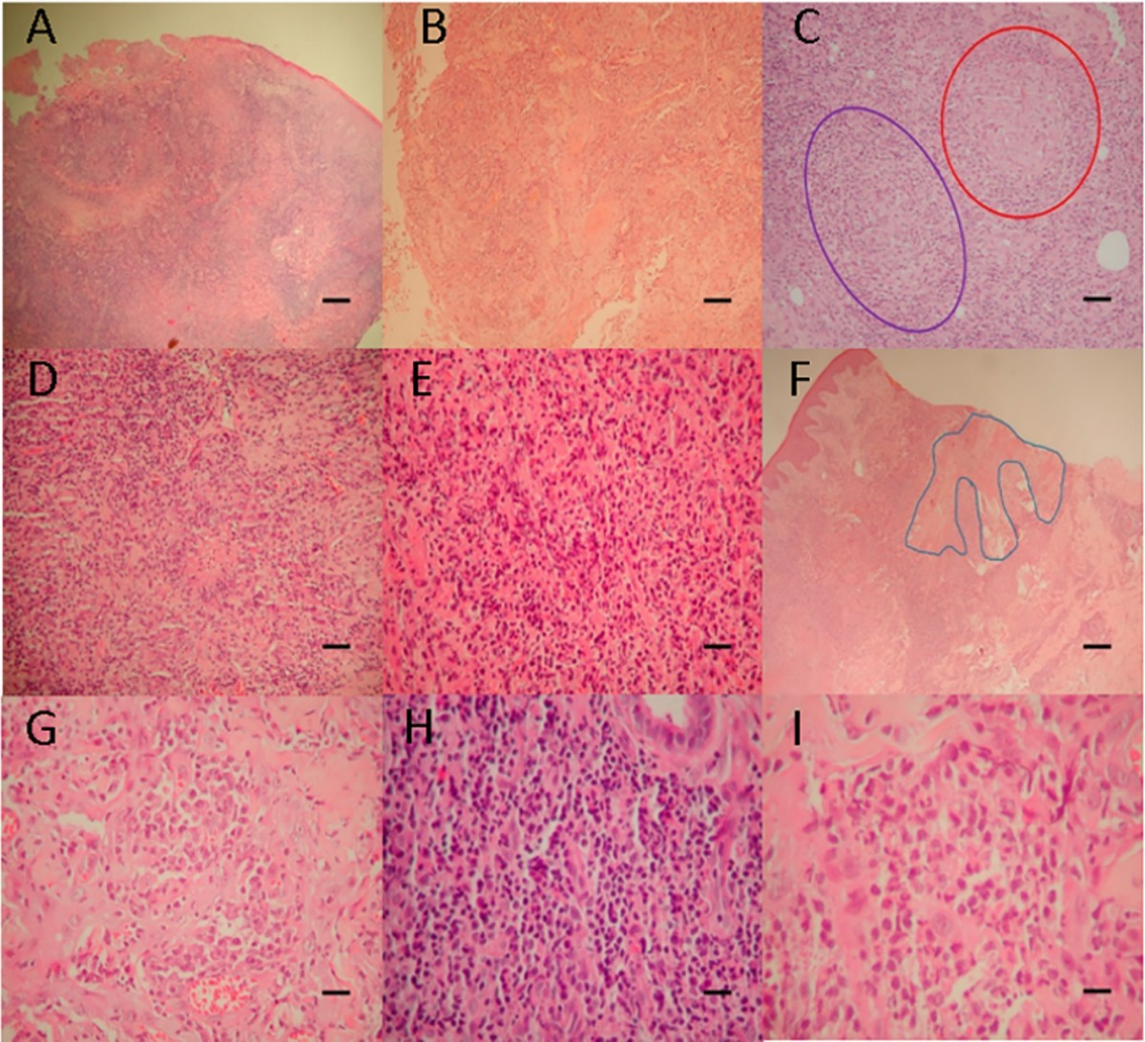
Images showing histopathological changes found in inner and outer edge of ulcerated skin lesions of 19 patients confirmed with CL, Rio de Janeiro, Brazil, 2009–2010. A: HE x 40. Well-characterized ulcer—loss of epithelial lining, bottom of the ulcer with fibrinous and necrotic material, and just below, granulation tissue (proliferation of vessels). B: HE x 100. Pseudoepitheliomatous hyperplasia. C: HE x 400. Malformed granuloma (purple ellipse on the left) and well formed (red circle on the right). D: HE x 100. Caseous necrosis in malformed granuloma. E: HE x 400. Liquefactive necrosis (= neutrophils ++; small abscesses). F: HE x 40. Fibrinoid necrosis (blue line). G: HE x 400. Plasmocytes +++, in the midst of pseudoepitheliomatous hyperplasia. H: HE x 400. Lymphocytes +++. I: HE x 400. Neutrophils + (isolated). (HE—hematoxylin-eosin). Scale bar = A: 250μm; B, D, F: 100μm; C, E, G, H, I: 50μm.

In the quantitative evaluation of amastigote forms, the parasite index was higher in the inner edge compared to the outer edge (*P* = 0.003), as well as the number of amastigote forms per field (*P* = 0.002) and the number of amastigote forms per vacuole (*P* = 0.006). The individual results of these samples can be seen in S2 Table. Different histopathological findings found in the samples are shown in Fig 5.

## Discussion

Here we evaluated comparatively parasite load by qPCR, quantification of amastigotes in lesion smears and histopathological findings in tissue fragments of inner and outer edges of ulcerated lesions of CL due to *L*.*(V*.*) braziliensis* infection.

Concerning the lesion sampling site, the scraping procedure on the ulcer inner edge showed a higher parasite load on the qPCR. Some authors have also demonstrated a higher parasite density of *Leishmania (Viannia)* species in the inner edge or even the center of the lesion in comparison to the outer edge [8–10].

In this study, it was possible to quantify DNA of all samples from lesion smears stored for about ten years, using a protocol based on the lysis of the material with proteinase K. Other studies using this protocol also obtained good results in the extraction of *Leishmania* DNA from clinical material from microscopy slides fixed and stained for several years [17–20].

It is worth mentioning that all the slides prepared with material from the ulcer inner edge of the lesion had a higher concentration of total DNA than the outer edge and imprint slides when quantified by fluorimetry. This fact suggests that it is possible to collect a greater amount of biological material on the ulcer inner edge than in the outer edge.

The choice of primers to be used in the qPCR reaction is an important factor on the success of the method. Although primers targeting to the small subunit ribosomal RNA gene (SSU rRNA) gene locus have better accuracy in relation to kDNA, they have less sensitivity due to a lower number of copies present in the *Leishmania* genome (ranging from 20 to 160) [21–24]. Due to the scarcity of clinical material coming from the slides, we used primers targeting to the conserved region of the kDNA minicircle, since this target has up to 10,000 copies per amastigote, guaranteeing a high sensitivity [25, 26]. Other studies that carried out the quantification of *Leishmania* parasite load also used this target [8, 10, 27].

The higher parasite load obtained by IE scraping in relation to the imprint is an important finding, considering that the imprint is a procedure that requires a biopsy, a surgical procedure, exclusive to the medical professional, implicating anesthesia and suture. Because of this, its application in endemic areas becomes unpractical. In this context, IE scraping is a simple, less invasive alternative that does not require anesthesia and can be performed by a non-medical professional in the diagnostic routine [11].

The imprint had a lower parasite load on the qPCR than scraping samples from the ulcer inner edge and similar to those of the outer edge, corroborating the results of other authors [10]. This lower parasite load could be explained by the way the tissue fragments used in the preparation of the imprint are obtained—by biopsy with a 5mm punch including 1/3 of ulcer inner edge and 2/3 of the outer edge of the lesion (clinical protocol–INI/FIOCRUZ). Another possible explanation would be the fact that the scraping on the inner edge of the lesion allows the collection of a sample from the superficial region of the dermis, which has been shown to contain a higher amount of parasite DNA in relation to the lower dermis and hypodermis [28]. The latter skin layers are included in the biopsy imprint, raising the proportion of human DNA vs. parasite DNA in that preparation [28, 29].

In respect to lesion evolution time, although we found higher parasite load median values on samples with ≤3 months lesions, when these results were statistically analyzed, no association was found between these parameters in disagreement some authors [27, 30]. It is important to notice that lesion evolution time was informed by the patients and was, therefore, subjective and early symptoms often go undetected.

Upon microscopic examination, the imprint also showed a smaller number of positive delimited areas when compared to IE. Paradoxically, in a former study the sensitivity of this method was higher than that of the IE scraping, as it allowed the diagnosis of a greater number of patients [11]. Sensitivity is an important parameter when choosing a diagnostic method. The higher sensitivity can also be explained due to the tissue samples used in the preparation being previously cleaned with sterile gauze to remove excess blood. This procedure could explain the easier visualization of the slides made by imprint when compared to the slides made by smear of material obtained by scraping. The latter usually have cellular debris and erythrocytes that make reading difficult, especially those slides made with material obtained by scarifying the outer edge of the ulcerated lesion [3, 11].

To our knowledge this is the first study that compares the inner and outer edges of CL ulcerated skin lesions by histopathological examination to investigate cell types, histopathological changes, and the number of amastigotes forms of *L*.(*V*.).*braziliensis*. In this study, we could distinguish the inner and outer edge in 19 slides from a total of 39. Such loss can also be explained by the method the biopsy was performed, with a 5mm punch, obtaining small tissue fragments.

The necrosis of the infected tissues is an important defense mechanism in the pathogenesis of CL [31, 32]. In our study, we could observe more cases of necrosis in the inner edge. These were mainly of the caseous and fibrinoid types. The presence of caseous necrosis in histological sections is strongly associated with tuberculosis, and may also be caused by some fungal diseases [32]. The presence of this type of necrosis in patients with CL could lead to a mistaken presumptive diagnosis, especially in samples with low parasite load, due to the difficulty in finding amastigote forms [33]. The presence of caseous necrosis in patients with CL has also been reported by other authors [31, 34].

Necrosis is defined as a process of elimination of amastigotes by the destruction of macrophages [35]. However, some authors relate the presence of necrosis with the presence of amastigotes [36]. In our study, the most significant presence of necrosis concomitantly with a higher parasite index was found on the ulcer inner edge.

Neutrophils have a dual function in the formation of infection, and they are the first effector cells of the innate immune system, *Leishmania* parasites can still survive this mechanism and multiply because amastigote forms are not completely destroyed when neutrophils start apoptosis and are phagocytosed by macrophages. For this reason, neutrophils have been called Trojan horses in *Leishmania* sp. infection [37]. In our study, neutrophils were found in a similar index in both evaluated lesion sites.

The number of lymphocytes and plasmocytes increases accordingly to the development of the lesion and the latter has been correlated with a higher number of amastigote forms [33]. In our study we observed a higher parasite index and a higher number of plasmocytes in the region of the inner edge of the lesion, corroborating with all other findings. One of the limitations of this study was the small number of samples evaluated by histopathological examination, due to the impossibility of making a distinction between the inner and outer edges in some preparations. Future studies of samples collected from the inner and outer edges independently should be made in order to deepen the knowledge of the disease pathogenesis and parasite distribution within the lesion.

The results presented here suggest that samples collected by scraping the inner edge of an ulcerated CL lesion are the most appropriate for diagnosis, both for molecular diagnosis and for direct parasitological examination.

## Supporting information

S1 Table Demographic data, results of parasite load by qPCR and number of positive delimited areas in the direct exam of 39 patients diagnosed with CL, seen in Rio de Janeiro, Brazil (2009–2010). (DOCX)

S2 Table Semi-quantitative histopathological evaluation of amastigote forms found in samples from inner and outer edge of histological sections of ulcerated skin lesions of 19 patients diagnosed with CL, seen in Rio de Janeiro, Brazil (2009–2010). (DOCX)
