# Supplementary material for: Comparison of parasite load by qPCR and histopathological changes of inner and outer edge of ulcerated cutaneous lesions of cutaneous leishmaniasis
Source: PLoS One. 2021 Jan 21;16(1):e0243978. doi: 10.1371/journal.pone.0243978 (PMC7819606; doi:10.1371/journal.pone.0243978)
Supplement: S1 Table — (DOCX) [file pone.0243978.s001.docx]

**S1 Table.** Demographic data, results of parasite load by qPCR and number of positive delimited areas in the direct exam of 39 patients diagnosed with CL, seen in Rio de Janeiro, Brazil (2009-2010).

| Patient code | Sex | Inner Edge | | Outer Edge | | Imprint | | Time of lesion evolution (months) | Number of lesions |
| --- | --- | --- | --- | --- | --- | --- | --- | --- | --- |
|  |  | Parasite Load | Nº of positive delimited areas | Parasite Load | Nº of positive delimited areas | Parasite Load | Nº of positive delimited areas |  |  |
| **1** | Male | 3,60E+05 | 12 | 1,32E+05 | 12 | 2,03E+01 | 12 | 2 | 1 |
| **2** | Male | 5,20E+02 | Negative | 1,00E+02 | Negative | 5.56E+04 | Negative | 2 | 1 |
| **3** | Female | 4,97E+04 | Negative | 3,53E+02 | Negative | 2,02E+02 | Negative | 1,5 | 1 |
| **4** | Male | 7,44E+05 | 11 | 1,44E+04 | 1 | 1.09E+05 | 8 | 2 | 1 |
| **5** | Male | 1,48E+05 | 12 | 1,81E+05 | 8 | 6,40E+02 | 3 | 2 | 1 |
| **6** | Female | 2,11E+02 | Negative | 3,56E+01 | Negative | 8,09E+01 | Negative | 5 | 2 |
| **7** | Male | 2,99E+03 | 3 | 1,37E+04 | Negative | 2,66E+03 | 2 | 2 | 8 |
| **8** | Female | 1,68E+03 | Negative | 9,36E+04 | Negative | 1,95E+02 | Negative | 2 | 1 |
| **9** | Male | 1,19E+03 | Negative | 1,67E+03 | Negative | 2,40E+02 | Negative | 2,5 | 3 |
| **10** | Male | 9,14E+05 | 11 | 2,56E+04 | 1 | 3,63E+05 | 12 | 1 | 1 |
| **11** | Male | 2,11E+05 | 12 | 9,93E+04 | 8 | 7,01E+03 | 1 | 0,5 | 1 |
| **12** | Female | 1,92E+02 | Negative | 1,11E+02 | Negative | 1,09E+03 | 2 | 3 | 8 |
| **13** | Female | 1,22E+06 | 12 | 1,05E+04 | 1 | 2,48E+05 | 5 | 5 | 1 |
| **14** | Female | 1,24E+03 | Negative | 1,15E+03 | Negative | 6,00E+02 | 1 | 5 | 1 |
| **15** | Female | 7,97E+03 | Negative | 3,57E+02 | Negative | 1,54E+04 | 9 | 2,5 | 3 |
| **16** | Male | 3,93E+04 | 1 | 1,59E+02 | Negative | 1,21E+04 | 1 | 3,5 | 1 |
| **17** | Male | 5,73E+05 | 12 | 6,63E+04 | 4 | 1,46E+05 | 4 | 1,5 | 1 |
| **18** | Female | 5,77E+06 | 10 | 4,41E+06 | 10 | 3,87E+05 | 3 | 6 | 4 |
| **19** | Male | 2,77E+06 | 12 | 3,65E+05 | 8 | 7,89E+05 | 11 | 2,5 | 1 |
| **20** | Female | 2,03E+06 | 6 | 8,61E+03 | Negative | 4,19E+05 | 10 | 1 | 1 |
| **21** | Male | 4,91E+04 | 4 | 7,54E+04 | 9 | 7,97E+04 | 12 | 1 | 1 |
| **22** | Male | 3,77E+03 | 1 | 8,26E+04 | 8 | 3,08E+03 | Negative | 5 | 3 |
| **23** | Female | 5,73E+04 | 12 | 3,87E+04 | 11 | 5,45E+03 | Negative | 6 | 1 |
| **24** | Male | 3,42E+06 | 12 | 6,39E+03 | Negative | 1,48E+05 | 8 | 1 | 1 |
| **25** | Male | 3,06E+05 | 1 | 6,46E+04 | 1 | 2,23E+05 | 7 | 2 | 1 |
| **26** | Male | 1,30E+02 | Negative | 8,91E+01 | Negative | 4,62E+01 | Negative | 4,5 | 1 |
| **27** | Female | 1,20E+03 | Negative | 4,47E+03 | Negative | 3,85E+01 | Negative | 1 | 2 |
| **28** | Male | 2,64E+05 | 4 | 2,70E+05 | 6 | 5,58E+04 | 6 | 2,5 | 3 |
| **29** | Male | 1,02E+05 | 1 | 2,57E+03 | Negative | 8,15E+04 | 3 | 2 | 1 |
| **30** | Male | 1,74E+00 | Negative | 5,93E+02 | Negative | 9,47E+02 | 1 | 2 | 3 |
| **31** | Male | 4,61E+03 | Negative | 9,47E+02 | Negative | 1,12E+02 | Negative | 3 | 44 |
| **32** | Male | 1,13E+06 | 11 | 2,10E+05 | 6 | 3,49E+05 | 7 | 3 | 1 |
| **33** | Female | 1,38E+02 | Negative | 2,74E+03 | Negative | 4,61E+01 | Negative | 3 | 1 |
| **34** | Male | 4,95E+02 | Negative | 6,98E+01 | Negative | 5,35E+03 | 2 | 1 | 5 |
| **35** | Male | 1,84E+02 | Negative | 1,55E+02 | Negative | 5,30E+03 | 1 | 1 | 1 |
| **36** | Male | 4,62E+03 | Negative | 6,80E+03 | Negative | 3,73E+03 | Negative | 1 | 3 |
| **37** | Male | 4,69E+04 | 8 | 4,29E+04 | 9 | 1,60E+04 | 9 | 3 | 1 |
| **38** | Male | 1,43E+05 | 12 | 1,63E+04 | 2 | 5,93E+03 | 1 | 53 | 1 |
| **39** | Male | 2,12E+05 | 9 | 3,51E+03 | Negative | 2,49E+05 | 8 | 7 | 1 |
