# Supplementary material for: Comparison of parasite load by qPCR and histopathological changes of inner and outer edge of ulcerated cutaneous lesions of cutaneous leishmaniasis
Source: PLoS One. 2021 Jan 21;16(1):e0243978. doi: 10.1371/journal.pone.0243978 (PMC7819606; doi:10.1371/journal.pone.0243978)
Supplement: S2 Table — (DOCX) [file pone.0243978.s002.docx]

**S2 Table.** Semi-quantitative histopathological evaluation of amastigote forms found in samples from inner and outer edge of histological sections of ulcerated skin lesions of 19 patients diagnosed with CL, seen in Rio de Janeiro, Brazil (2009-2010).

| **Patient code** | **Inner Edge** | | | **Outer Edge** | | | |
| --- | --- | --- | --- | --- | --- | --- | --- |
|  | **Parasite index** | **Amastigote per field** | **Amastigote inside** **parasitophorous vacuole** | | **Parasite index** | **Amastigote per field** | **Amastigote inside** **parasitophorous vacuole** |
| **1** | 1+ | 1 | 0 | | 0 | 0 | 0 |
| **2** | 4+ | 53 | 7 | | 3+ | 28 | 5 |
| **3** | 1+ | 3 | 1 | | 0 | 0 | 0 |
| **4** | 0 | 0 | 0 | | 0 | 0 | 0 |
| **5** | 3+ | 49 | 6 | | 0 | 0 | 0 |
| **6** | 3+ | 18 | 2 | | 1+ | 1 | 1 |
| **7** | 4+ | 66 | 7 | | 0 | 0 | 0 |
| **8** | 2+ | 4 | 1 | | 1+ | 1 | 1 |
| **9** | 1+ | 2 | 1 | | 1+ | 2 | 1 |
| **10** | 4+ | 136 | 14 | | 0 | 0 | 0 |
| **11** | 3 | 14 | 2 | | 1+ | 1 | 1 |
| **12** | 3+ | 15 | 4 | | 3+ | 11 | 2 |
| **13** | 3+ | 21 | 4 | | 2+ | 6 | 2 |
| **14** | 0 | 0 | 0 | | 0 | 0 | 0 |
| **15** | 3+ | 50 | 5 | | 0 | 0 | 0 |
| **16** | 0 | 0 | 0 | | 1+ | 2 | 1 |
| **17** | 0 | 0 | 0 | | 0 | 0 | 0 |
| **18** | 0 | 0 | 0 | | 0 | 0 | 0 |
| **19** | 3+ | 15 | 1 | | 1+ | 1 | 1 |
